# Notes and News

**Published:** 1891-10-24

**Authors:** 


### Notes and News.

COLLECTED AND COMMUNICATED.

The following memorial was circulated amongst the Trustees of the Royal Infirmary, Manchester, for signature, to be duly presented to the Board of Management: "Gentlemen,—We understand that a proposition for the enlargement of the Manchester Royal Infirmary on the present site is now under your consideration, and that plans for this enlargement will shortly be submitted for our acceptance as trustees. We are of opinion that whilst it is manifestly important that the Infirmary should be retained on its present site, in the utmost state of efficiency, any extension of the accommodation thereon is very undesirable.—We are, yours faithfully." The Board have obtained knowledge of this before its presentation, and protest against any opinion being formed as to the desirability of extension or not until they have submitted their properly drawn up proposals and plans for consideration. The Secretary to the memorialists replies that "the desire is to avert a conflict that could not fail to be very injurious to the best interests of the Royal Infirmary, by conveying to the Board before they go to the trouble and expense of having plans prepared the strong feeling that exists against any extension of the building on the present site."

At the Quarterly Court of the Addenbrooke's Hospital, just held, the Committee of Management and Medical Staff proposed that the vacancy on the surgical staff should be filled up by the appointment of an assistant surgeon in the place of a full surgeon, and an amendment was carried to the effect that in future the surgical staff should consist of four surgeons and an assistant surgeon. A poll is to be taken on the question. Dr. Donald Macalister proposed the resolution, and, on the amendment being carried, called for a poll. As has been seen, the meeting was not unanimous in favour of the alterations suggested, and the discussion is now being carried on in the papers. One Life Governor who is against extending the number of the staff, points out that there is no first-class hospital in England in which so few patients are divided among so many surgeons as at Addenbrooke's. There are between fifty and sixty surgical beds constantly occupied, and four surgeons to attend the patients. In a large London hospital each surgeon has between forty and fifty patients under his care. He also asserts that this increase in number will decrease the value of an appointment at the hospital by lessening the experience to be obtained there. The objections do not apply to the election of an assistant surgeon or surgeons, but to a large staff of full surgeons. Mr. Wallis is the outgoing surgeon who will not seek re-election. The funds of the hospital show no improvement.

We have received, through the courtesy of the Secretary of the Glasgow Royal Infirmary, a printed copy of the report of the "Committee appointed by the managers to inquire into a complaint in relation to the food and hours of work of the nurses." The report is too long for us to publish in full, and it has reached us too late for detailed consideration this week. But we propose to "take it to avizandum," as the Scotch judges say, and to deal with it hereafter as fully as the nature of the case may seem to require.

Dr. Collie's resignation was again the principal topic of discussion at the Saturday meeting of the Metropolitan Asylums Board, but a settlement of the difficulties which have arisen out of what was for long known as the Eastern Hospital Scandal seems as far off as ever. Dr. Collie, it was reported, had written to the Local Government Board traversing certain points in the decision of the Inspector, and asking whether, under the circumstances, they desired him to send in his resignation without further communication. The only reply was a practical confirmation of the former decision. Several resolutions and amendments were proposed by various members, some of them of a dilatory character, and others demanding the resignation of Dr. Collie, not out of any ill-feeling against that gentleman, but simply because their duty to the poor in the hospital required such a course. The majority, however, seemed undecided what to do, negatived all the motions submitted to them, and at last adjourned without doing anything.

The annual Report of the Governesses' Convalescent Home at Southport is just out. Such institutions are of much value to a class of the community who frequently greatly need help, and whom it is not always easy to reach. We are, therefore, glad to see that the Home in question is in a flourishing condition. The accounts show a balance in hand, although many necessary improvements and alterations have been effected. There has been a large increase of new visitors, testifying to the popularity of the institution, as the accommodation is not entirely free. The Home has lost a kind friend in Mrs. Stead, who was a member of the original Committee, and a staunch and generous helper during the twenty-five years of its existence. We think some of our readers may be glad of the suggestion that gifts of clothing, papers, &c., are gratefully received. Many ladies are at a loss sometimes to know how to dispose of apparel unsuited for distribution amongst the poor, and for which they themselves have no use.

Last week the foundation-stone of an hospital building, which is certain to prove a valuable adjunct to the Stirling District Asylum, was formally laid by Colonel Stirling, of Tarduf, Chairman of the Board of Directors. The new hospital block is being erected to the north of the existing buildings. The situation is at once convenient and cheery. The total length of the building is close upon 100 yards, and the greatest breadth 175 feet. The salient features of the design are a central block and two side wings, with connecting corridors. The central block contains the administrative department, with medical office, surgery, Matron's room, and waiting-rooms for visitors. The domestic offices are placed here, and the bed-rooms for nurses and attendants on the upper floor. The dining-hall is a large and lofty apartment. Only the wings of the central block and the administrative department rise two storeys high. There are single rooms for acute cases, and special provision is made here for isolation. The heating will be by steam pipes beneath the main corridor, and fresh air will be admitted to the various wards after being heated by coming into contact with these pipes; open fireplaces are also provided in the large dormitories and day-rooms. The buildings have been designed in the Elizabethan style. Messrs. A. and W. Black, Falkirk, are the architects.

